# Five-year study of the effects of simulated nitrogen deposition levels and forms on soil nitrous oxide emissions from a temperate forest in northern China

**DOI:** 10.1371/journal.pone.0189831

**Published:** 2017-12-18

**Authors:** Ke Xu, Chunmei Wang, Xintong Yang

**Affiliations:** 1 College of Environmental Science and Engineering, Beijing Forestry University, Beijing, China; 2 Beijing Solid Waste Treatment Co., Ltd., Beijing Environmental Sanitation Engineering Group Co., Ltd., Beijing, China; Institute of Tibetan Plateau Research Chinese Academy of Sciences, CHINA

## Abstract

Few studies have quantified the effects of different levels and forms of nitrogen (N) deposition on soil nitrous oxide (N_2_O) emissions from temperate forest soils. A 5-year field experiment was conducted to investigate the effects of multiple forms and levels of N additions on soil N_2_O emissions, by using the static closed chamber method at Xi Mountain Experimental Forest Station in northern China. The experiment included a control (no N added), and additions of NH_4_NO_3_, NaNO_3_, and (NH_4_)_2_SO_4_ that each had two levels: 50 kg N ha^−1^ yr^−1^ and 150 kg N ha^−1^ yr^−1^. All plots were treated to simulate increased N deposition on a monthly schedule during the annual growing season (March to October) and soil N_2_O emissions were measured monthly from March 2011 to February 2016. Simultaneously, the temperature, moisture, and inorganic N contents of soil were also measured to explore how the main factors may have affected soil N_2_O emission. The results showed that the types and levels of N addition significantly increased soil inorganic N contents, and the accumulation of soil NO_3_^–^-N was significantly higher than that of soil NH_4_^+^–N due to N addition. The three N forms significantly increased the average N_2_O emissions (*P* < 0.05) in the order of NH_4_NO_3_ > (NH_4_)_2_SO_4_ > NaNO_3_ by 355.95%, 266.35%, and 187.71%, respectively, compared with control. The promotion of N_2_O emission via the NH_4_^+^–N addition was significantly more than that via the NO_3_^–^–N addition, while N addition at a high level exerted a stronger effect than at the low-level. N addition exerted significantly stronger effects on cumulative N_2_O emissions in the initial years, especially the third year when the increased cumulative N_2_O emission reached their maximum. In the later years, the increases persisted but were weakened. Increasing inorganic N concentration could change soil from being N-limited to N-rich, and then N-saturated, and so the promotion on soil available N effect increased and then decreased. Moreover, the soil NH_4_^+^–N, NO_3_^–^-N, temperature, and water-filled pore space were all positively correlated with soil N_2_O emissions. These findings suggest that atmospheric N deposition can significantly promote soil N_2_O emission, and that exogenous NH_4_^+^–N and NO_3_^–^-N inputs into temperate forests can have synergic effects on soil N_2_O emission. In future research, both aspects should be better distinguished in the N cycle and balance of terrestrial ecosystems by using ^15^N tracer methods.

## Introduction

Nitrous oxide (N_2_O) is not only a potent greenhouse gas whose global warming potential is 298- and 21-fold that of CO_2_ and CH_4_, but it also contributes to stratospheric ozone depletion [1]. Emissions of N_2_O from soil have been identified as the primary source (57%) of total global N_2_O emissions [2].

**Fig 1:**
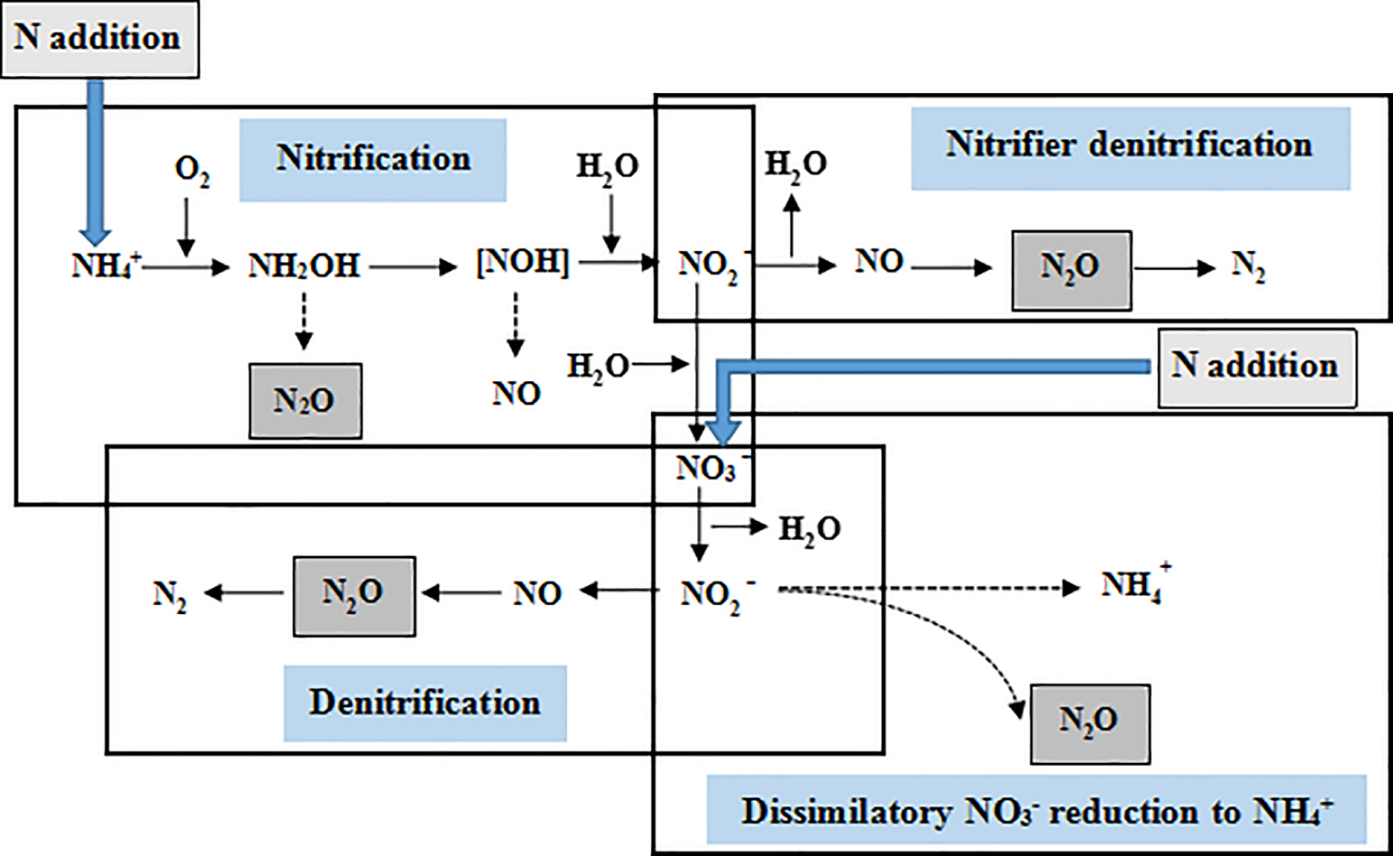
Main processes produce N_2_O in soils.

Nitrification and denitrification are the two main processes that produce N_2_O in soils and both can occur simultaneously (Fig 1). N_2_O is produced by denitrifying bacteria during the reduction of NO_3_^–^ or NO_2_^–^ to N_2_O and N_2_, or released as an intermediate product when nitrifying bacteria oxidize NH_4_^+^–N to NO_3_^–^ and NO_2_^–^ [3]. These two processes may be affected by soil water content, temperature, N availability and pH, as well as other particular biotic or abiotic properties [4–6]. Inorganic N is a key factor regulating soil N_2_O emission [4–5, 7–8] (Fig 1). In general, increasing available mineral N in soils leads to enhanced N_2_O formation and emission via increased nitrification and denitrification rates [9]. Soil N_2_O emission is also driven by soil temperature and water content [10]. Some previous studies indicated soil N_2_O emissions were increased under conditions of higher soil water content and soil temperature [10]. The latter may regulate soil N_2_O emission by influencing N_2_O-producing microorganisms, such as nitrifying and denitrifying bacteria [11]. Furthermore, low soil moisture can reduce the temperature sensitivity of soil microbes, so that the diffusion of extracellular enzymes in the substrate are lowered [12].

China is now ranked third behind Europe and North America in terms of the scale of anthropogenic reactive N emissions, and has been experiencing a dramatic increase in anthropogenic reactive N due to its rapid economic development [10]. The average N deposition in our study area was 13.2 kg N ha^−1^ yr^−1^ in the 1980s and 21.1 kg N ha^−1^ yr^−1^ in the 2000s [13]. Alongside increases in N deposition there have been decreases in the ratio of NH_4_^+^–N to NO_3_^−^-N deposition, from approximately 5 to 2, from the 1980s to the 2000s, although NH_4_^+^–N remains the dominant form of N deposition [13]. Nationally, N deposition is a more serious issue in the north compared with the other regions of China [14].

Increasing N deposition could influence the production and emission of N_2_O by disturbing the balance between microbial N mineralization and immobilization, with the consequences for the relative availability of soil NH_4_^+^–N and NO_3_^−^-N [15] (Fig 1). Most studies report that raising N addition levels could linearly stimulate soil N_2_O emissions [4–5, 16–17]. A meta-analysis of global N addition experiments showed that N additions increased soil N_2_O emissions by an average of 134% in terrestrial ecosystems [18]. Some plausible mechanisms have been proposed to clarify the promotion effect of N addition for soil N_2_O emissions: (1) Without additional N, the N retention in soil is mainly used by plants and microorganisms to maintain biomass and growth, so less N becomes lost as gaseous N [19]; (2) The amount of additional N greatly exceeds the atmospheric N deposition, thus leading to N accumulation in forest soil, which can benefit nitrifying and denitrifying bacteria [20] which would stimulate the nitrification rate and N_2_O emissions [21]. However, some studies indicated that N addition has no significant effect on soil N_2_O emission, which might be attributed to particular N addition threshold level for increased N_2_O emissions [7, 22]. Thornton and Valente [23] found that the increased rate of soil N_2_O emissions was low at high N-addition levels; this may have occurred because the high level N addition to soil drove other limitations, such as carbon availability, thereby decreasing the C/N ratios that regulate the status of N saturation, which likely had a strong influence on N_2_O emission [24]. Furthermore, some studies have shown denitrification to be the main source of soil N_2_O emissions [25–26], whereas other studies reported that nitrification were primarily responsible for soil N_2_O emissions [7, 27–28]. Clearly then, how soil N_2_O emissions respond to additional N appears to be inconsistent.

The NH_4_^+^–N/NO_3_^–^–N ratio showed a decreasing trend in our study area [29], and so clarifying the response of soil N_2_O emission to different forms and levels of N addition now is necessary. However, several previous studies that stimulated N deposition only considered NH_4_NO_3_ [2, 4, 6], while others that did examine N deposition in varied N forms only reported their short-term effects on N_2_O emission [30]. In addition, some studies have focused on soil core incubations in the laboratory [31], which are conditions that differ greatly from those in the field. Therefore, from both a scientific and management perspective, further examination of the characteristics of different levels and forms of N addition is critically important for better understanding how N deposition affects soil N_2_O emissions in temperate forest soils.

In our study, we report the results of continuous measurements of soil N_2_O emissions over a 5-year period from a temperate forest in northern China. Based on the above analysis, we hypothesized that (1) N addition could increase soil N_2_O emission and that this promotion effect likely increased with the N addition level; (2) Applying NO_3_^−^ and NH_4_^+^-N in combination could promote soil N_2_O emission more than would their respective single applications.

## Materials and methods

### Study area

The study was conducted in a temperate forest of the Xi Mountain Experimental Forest Station (31°54′32″ N, 110°68′08″ E, 133 m a.s.l.) in Beijing, northern China. The station belongs to Beijing Forestry University. The study area is characterized by a temperate continental monsoon climate with a maximum air temperature of 31°C in July and a minimum of –9°C in January. Mean annual temperature is 11.6°C and the average annual precipitation is 630 mm. During the 5-year experimental period, the yearly maximum and minimum temperatures were, respectively, 31, 31, 32, 33, 31°C and –9, –8, –8, –5, –5°C, while the total precipitation received annually was 721, 759, 508, 500, and 459 mm. At this research station, *Quercus liaotungensis* is the zonal vegetation with an average age of 62 years. The diameter at breast height, canopy closure, average height, and density were 9.7 cm, 69%, 8.4 m, and 2963 trees ha^−1^. The soil here is classified as Chromic Luvisols (WRB Soil Classification) composed of 51% sand, 40% silt, and 9% clay. The thickness of the soil humus horizon (A horizon) is approximately 3−5 cm, and the O horizon thickness < 3 cm. Before starting the experiment, soil samples from the upper 10 cm of soil in each plot (with three replicates) were collected by using corers in March 2011. Initial soil properties were measured and showed no significant differences among the plots (Table 1).

**Table 1:** Soil properties of the sampling area.

	Treatment						
Variable	Control	NaNO_3_		(NH_4_)_2_SO_4_		NH_4_NO_3_	
		L	H	L	H	L	H
pH	7.18±0.28a	7.02±0.39a	7.16±0.49a	7.01±0.35a	7.20±0.28a	7.19±0.39a	7.12±0.21a
Organic C (g kg^-1^)	29.97±0.88a	30.14±1.45a	29.45±1.73a	28.37±1.65a	29.15±0.83a	30.33±1.38a	31.06±1.67a
Total N (g kg^-1^)	2.43±0.68a	2.35±0.84a	2.36±0.72a	2.40±0.68a	2.54±0.60a	2.39±0.66a	2.44±0.48a
NH_4_^+^-N (mg/kg)	2.82±0.20a	2.51±0.46a	2.37±0.43a	2.45±0.22a	2.41±0.36a	2.47±0.44a	2.55±0.36a
NO_3_^-^-N (mg/kg)	11.83±1.01a	12.80±1.02a	12.87±1.18a	13.05±1.09a	12.78±1.26a	13.06±1.22a	13.15±1.36a

L: 50 kg N ha^−1^ yr^−1^; H: 150 kg N ha^−1^ yr^−1.^

Treatments with same letter mean no significant difference in the whole row parameters.

### Experimental design

The experiments were performed from March 2011 to February 2016. Seven 10 m × 10 m N addition plots, with three replicates each (n = 21 plots in total), were randomly established and distributed on a flat ground dominated by the *Quercus liaotungensis* community at the research station. To ensure plot independence, 1.5-m buffer strips were set up between adjacent plots. As deposition of NH_4_^+^–N and NO_3_^–^–N showed great variation from month to month in the study area [30], three N-addition forms, namely NaNO_3_, (NH_4_)_2_SO_4_, and NH_4_NO_3_, were used to simulate the effects of deposited NH_4_^+^–N, NO_3_^–^–N, and their combination. According to the current level of atmospheric N deposition (30.6 kg N ha^–1^ yr^–1^) at the experimental site [30], two N-addition levels referred to as low N (L: 50 kg N ha^–1^ yr^–1^) and high N (H: 150 kg N ha^–1^ yr^–1^) were used to simulate a future increase in atmospheric N deposition by 1.5-fold and 5-fold. A control (0 kg N ha^–1^ yr^–1^) was used to calculate the net effect of naturally occurring N addition to the soil. From 2011 to 2015, additional N was evenly sprayed on the soil surface in plots by using sprayers, with eight equal applications made from March to October (i.e., the growing season). If it rained, the scheduled N addition was postponed to 1 day after the rain day. To reduce the effect of additional water on the experiment, control plots received an equivalent deionized water treatment.

### Gas sampling and measurement

Soil N_2_O emission measurements were performed three times in the first week of each month, from March 2011 to February 2016. Soil N_2_O emissions were measured using a static closed opaque chamber and gas chromatography method [32]. The chamber was made of stainless steel and consisted of a fixed base and a removable top (without bottom, length × width × height = 50 cm × 50 cm × 50 cm). Before measurement, the base, which supported the sampling chamber, was installed into the soil at a depth of 20 cm for the entire experiment to avoid soil disturbance. Soil temperatures were measured in each plot at a depth of 5 cm nearby the chamber before and after collecting gas samples. And the average temperature value was used for emission calculation. The fixed base frame was free of vegetation. When collecting the gases, we inserted the removable top into the fixed base. The chamber was covered with thermal insulation cotton to reduce the impact of direct radiative heating in the chamber and a digital thermometer in the chamber was used to record its air temperature. Two fans were used to increase mixing and uniformity of air in the chamber.

Gas samples were collected three times, from a sampling outlet at the top of the chamber, from 09:00 to 11:00 AM. local time on the first, fourth, and seventh day after N addition in each month from March 2011 to February 2016. If unpredicted extreme weather occurred, such as heavy rain or snow, this gas sampling was rescheduled. Gas samples were taken using 100 mL plastic syringes at intervals of 0, 10, 20, and 30 minutes after closing the chamber and inserting polyethylene-coated aluminum bags for soil N_2_O concentration analysis. Gas samples were analyzed within 6 h in a gas chromatograph (Agilent 7890A, Agilent Technologies Inc., Palo Alto, CA, USA) [33].

Soil N_2_O emissions were calculated as follows [33]:

$$F_{N_{2}O}=D\times H\times (\Delta c/\Delta t)$$ (1)

where, $F_{N_{2}O}$ refers to N_2_O emission (μg m^–2^ h^–1^); D refers to the gas density of the chamber (mol m^–3^); D = WP/RT; W refers to the molar mass of N_2_O (g mol^–1^); P refers to air pressure (Pa); T refers to the air temperature inside the chamber (K); R refers to the gas constant (J mol^–1^ K^–1^); H refers to the height of the sampling chamber (m); and Δc/Δt denotes the linear slope of the concentration change over the measurement period.

Soil cumulative N_2_O emissions were calculated by interpolating the N_2_O emissions measured between sampling periods [34]. Cumulative N_2_O emissions were calculated spanning the time period from March to February next year as follows [35]:

$$Cumulative\;N_{2}O\;emissions=\frac{\sum_{i=1}^{n}0.5\times (F_{i}+F_{i+1})\times (t_{i+1}-t_{i})\times 24}{100000}$$ (2)

where, F is the N_2_O emissions (μg m^–2^ h^–1^); *i* is the sampling number, i.e., samples collected in March had a value of 1 and those collected next February had a value of 12; and t is the sampling time based on the Julian day.

### Soil sampling and measurement

Considering that N_2_O release mainly occurred in the mineral horizon, litter was first removed from the soil surface (O horizon < 3 cm) when sampling the soil. Soil samples at 0–10 cm depth were collected from near the static chambers monthly. Soil samples were passed through a 2-mm sieve to remove roots, gravel, and stones for soil analyses. Part of the fresh soil was used for soil NH_4_^+^–N and NO_3_^–^–N content analyses, while the remaining portion was air-dried for pH measurement. Soil NO_3_^–^–N and NH_4_^+^–N concentrations were determined by the KCl extraction method [5]. Soil water content (WC) was measured using the standard oven-drying method at 105°C for 8 h. Bulk density (BD) was determined by the core method. Water-filled pore space (WFPS) (%) was calculated based on the equation:

$$WFPS=(WC\times BD)\times \frac{100}{\left(1-BD/2.65\right)}$$ (3)

where 2.65 (g cm^–3^) refers to the assumed soil particle density.

### Statistical analysis

All statistical analyses were conducted by SPSS v22.0 (IBM Corp., Armonk, USA) and the significance level for all statistical tests was set at *P* = 0.05. The differences in initial soil properties between different N-addition plots were examined using one-way analysis of variance (ANOVA) and least significant difference (LSD). Repeated-measures ANOVA was used to analyze the effects of N forms, N levels, experimental years, and their interactions on the temporal variation of soil N_2_O emissions, annual cumulative N_2_O emissions, ST, WFPS, and inorganic N concentrations. We examined the differences in annual N_2_O emissions within each single year among the N additions by one-way ANOVA and LSD testing, and the differences within each N addition throughout the 5 years. Pearson's correlation analyses and linear regression analyses were used to examine the relationships between soil N_2_O emissions and environmental variables. Means and standard deviations of N_2_O emissions were calculated, and the plot values represented means (n = 3) ± standard error (SE).

## Results

### Soil N_2_O emissions under N addition

**Fig 2:**
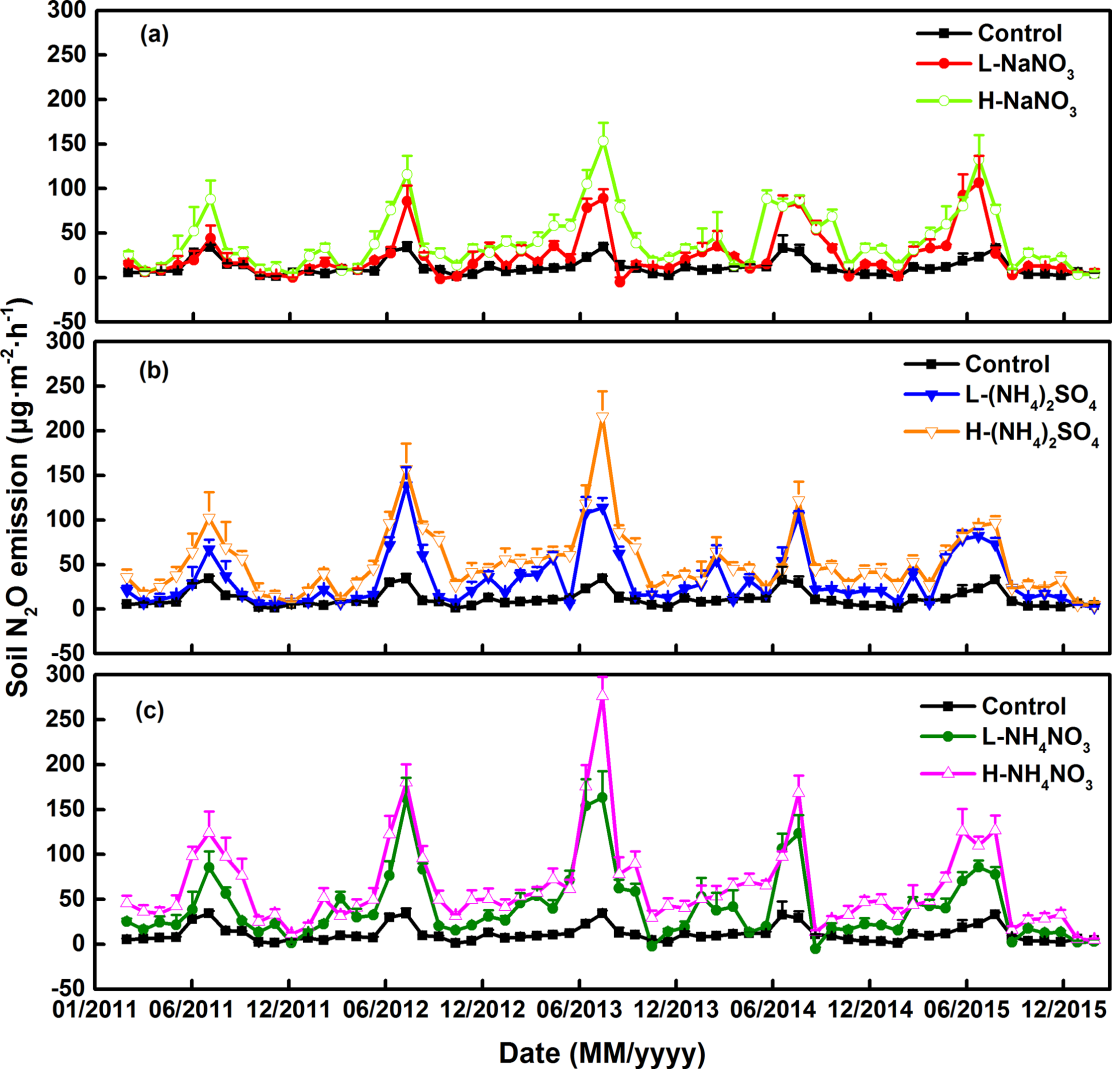
Variations of soil N_2_O emissions applied with different forms and levels of N addition among five–year experimental period. L: 50 kg N ha^–1^ yr^–1^; H: 150 kg N ha^–1^ yr^–1^. Error bars indicate the standard error of the mean (n = 9).

During the 5-year experimental period, the temperate forest soil was a net source of N_2_O. Soil N_2_O emissions were higher between May and September, but the values were lower and leveled off in other times of each year. Meanwhile, the peak of soil N_2_O emissions was concentrated in August of each year (Fig 2). Soil N_2_O emissions were significantly influenced by N forms, N levels, and the sampling time (*P* < 0.01), but the interaction effect of N forms and levels, months and N levels or months, N forms and N levels, did not significantly influence the soil N_2_O emissions (*P* > 0.05, Table 2).

**Table 2:** Summary of repeated measures ANOVA results (*F* values) indicating the effects of different forms and levels of N addition and experimental time on temporal variation of soil N_2_O emissions and annual cumulative N_2_O emissions.

N_2_O emissions			Cumulative N_2_O emissions		
Subjects	d.f.	*F*	Subjects	d.f.	*F*
**Between subjects**					
Form	2	8.367 **	Form	2	57.816 **
Level	1	24.979 **	Level	1	172.416 **
Form × Level	2	0.165 ns	Form × Level	2	1.138 ns
**Within subjects**					
Month	59	28.630 **	Year	4	120.249 **
Month × Form	118	3.774 **	Year × Form	8	19.198 **
Month × Level	59	2.040 ns	Year × Level	4	10.061 **
Month × Form × Level	118	0.624 ns	Year × Form × Level	8	2.571 *

* *P* <0.05

** *P* <0.01 and ns *P* > 0.05.

### Promotion effects of different N forms and levels

Different levels and forms of N addition and experimental time all significantly influenced the soil N_2_O emissions (*P* < 0.01, Table 2). As for the two N-level addition treatments, the N-addition treatments significantly increased soil N_2_O emissions, and this promotion effect was enhanced as the N-addition levels increased (Table 2, Fig 2). Soil N_2_O emissions ranged from 1.30 μg m^–2^ h^–1^ to 34.44 μg m^–2^ h^–1^, with an average value of 11.55 μg m^–2^ h^–1^ in the control plots (Fig 2). Compared to the control, the average N_2_O emissions in the low- and high-level N addition plots significantly increased by 186.02% and 353.98%, respectively. The maximal emissions were obtained in August 2013 for the low and high nitrogen addition serials, which were 163.23 and 276.33 μg m^–2^ h^–1^ in the L-NH_4_NO_3_ and H-NH_4_NO_3_ addition plots respectively, for all the three added nitrogen forms (Fig 2).

As for the N-addition treatments using the different forms of N, soil N_2_O emissions were significantly increased by NH_4_NO_3_, (NH_4_)_2_SO_4_, and NaNO_3_ additions in the order of NH_4_NO_3_ > (NH_4_)_2_SO_4_ > NaNO_3_ > control for the same level of N addition (Fig 2, Table 2). Compared to the control, the average N_2_O emissions in the NH_4_NO_3_, (NH_4_)_2_SO_4_, and NaNO_3_ addition plots significantly increased by 355.95%, 266.35%, and 187.71%, respectively (Fig 2). There was no significant interaction between N form and N level on soil N_2_O emissions (*P* > 0.05, Table 2).

### Interannual soil cumulative N_2_O emissions under N addition

Except for the interaction between N form and N level, year, N form and N level as well as all their interactions exerted significant effects on cumulative N_2_O emissions (Table 2). In the control plot, cumulative N_2_O emissions showed no significant differences among the 5 years (Table 3). However, in the N addition plots, the promotion effect of additional N on soil N_2_O emissions increased over time in the initial years, but then it decreased. As for the three N-form additions, ANOVA showed that the annual emissions were basically elevated by NH_4_NO_3_, (NH_4_)_2_SO_4_, and NaNO_3_ additions in the order of NH_4_NO_3_ > (NH_4_)_2_SO_4_ > NaNO_3_ > control (*P* < 0.05), but no significant differences were found between NaNO_3_ and control plots in the first year (Table 3, *P* > 0.05).

**Table 3:** Cumulative N_2_O emission (kg N ha^–1^ yr^–1^) from different N addition treatments plots.

Treatments	Cumulative N_2_O emissions (kg N ha^–1^ yr^–1^)				
	2011	2012	2013	2014	2015
Control	0.98±0.02_a(a)_	1.01±0.09_a(a)_	1.08±0.08_a(a)_	1.04±0.15_a(a)_	0.97±0.14_a(a)_
L-NH_4_NO_3_	2.52±0.03_d(a)_	4.28±0.34_d(d)_	5.32±0.43_d(e)_	3.18±0.10_c(c)_	2.88±0.04_b(b)_
H-NH_4_NO_3_	4.73±0.04_f(a)_	5.82±0.75_d(b)_	7.52±0.77_f(c)_	5.22±0.46_e(b)_	4.59±0.74_d(a)_
L-(NH_4_)_2_SO_4_	1.69±0.15_b(a)_	3.16±0.16_c(b)_	3.84±0.54_c(c)_	2.75±0.36_bc(b)_	2.86±0.23_b(b)_
H-(NH_4_)_2_SO_4_	3.41±0.00_e(a)_	5.25±0.24_e(c)_	6.21±0.25_e(d)_	4.13±0.48_d(b)_	3.71±0.28_c(b)_
L-NaNO_3_	1.15±0.18_a(a)_	1.89±0.20_b(b)_	2.57±0.23_b(c)_	2.65±0.25_b(c)_	2.63±0.32_b(c)_
H-NaNO_3_	2.31±0.17_c(a)_	3.30±0.05_c(b)_	4.97±0.73_cd(c)_	3.95±0.28_d(c)_	3.67±0.69_c(b)_

L: 50 kg N ha^–1^ yr^–1^; H: 150 kg N ha^–1^ yr^–1^.

Different superscripts of lowercase letters outside the parentheses indicate the significant differences at the level of *P* < 0.05 between the treatments within the same column, inside the parentheses indicate difference at the level of *P* < 0.05 between the experimental years within the same row.

### Environmental variables and their correlation with N_2_O emissions

**Fig 3:**
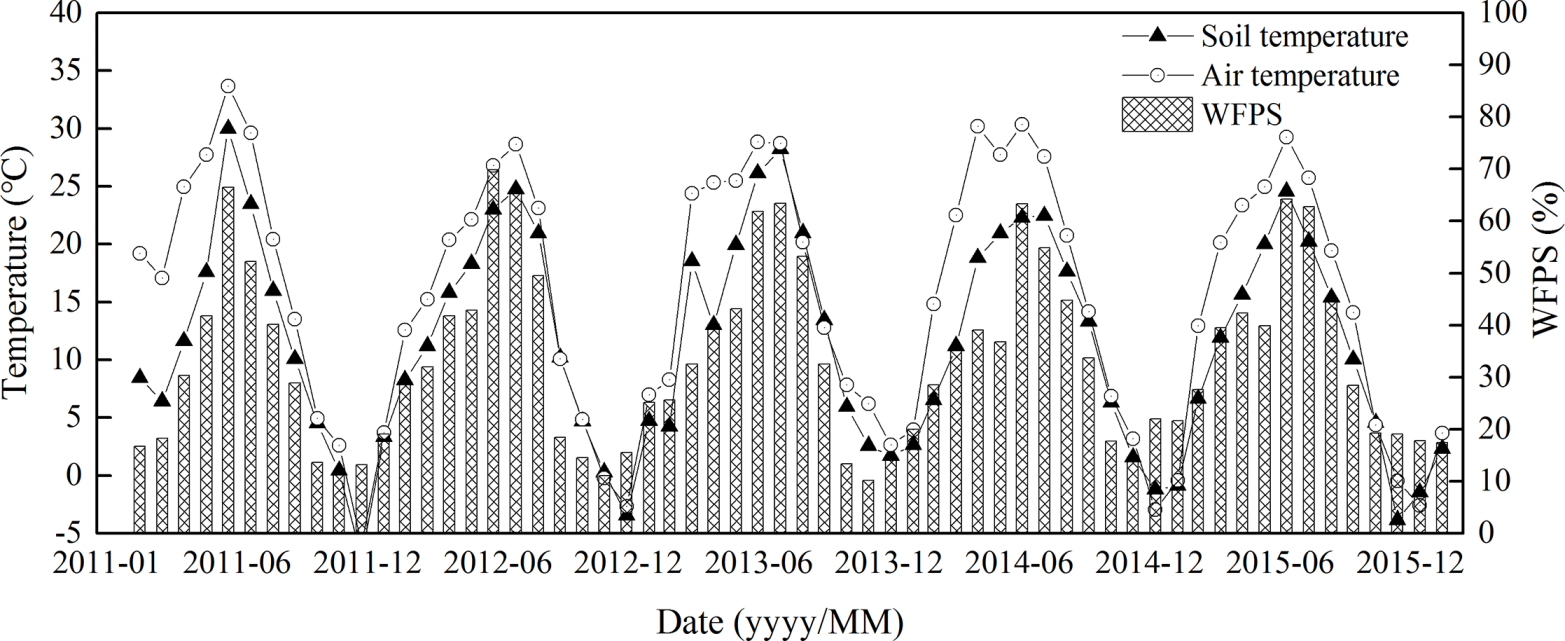
Water filled pore space (WFPS), soil temperature and air temperature in the observed period.

During the 5-year period, air temperature had a clear seasonal pattern with higher temperatures in wet seasons (May to September) and lower in dry seasons (November to February). Soil temperature (ST) at the 5-cm depth fluctuated greatly, following changes in air temperature. The highest ST was 29.9°C and lowest was –7.2°C. WFPS ranged from 10.20% to 69.84% and fluctuated greatly (Fig 3). There were no significant differences among different N-addition plots on ST and WFPS (*P* > 0.05, Table 4).

**Table 4:** Summary of repeated measures ANOVA results (F values) indicating the effects of different forms and levels of N addition and experimental time on soil temperature at 5 cm soil depth (ST), water-filled pore space (WFPS), and the concentrations of soil inorganic N (NO_3_^−^ and NH_4_^+^).

		ST		WFPS		NO_3_^−^		NH_4_^+^	
	d.f.	*F*	*P*	*F*	*P*	*F*	*P*	*F*	*P*
**Between subjects**									
Form	2	0.054	0.947	0.082	0.921	3.096	0.051	6.928	**0.002**
Level	1	0.000	0.996	0.004	0.947	7.633	**0.007**	1.680	0.199
Form×Level	2	0.020	0.981	0.011	0.989	0.008	0.992	0.273	0.762
**Within subjects**									
Date	59	6.498	**< 0.001**	39.766	**< 0001**	10.772	**< 0.001**	4.253	**0.002**
Date×Form	118	0.542	0.824	0.251	0.980	1.740	0.089	1.158	0.325
Date×Level	59	0.422	0.793	0.249	0.910	1.289	0.274	0.630	0.641
Date×Form×Level	118	0.358	0.942	0.609	0.770	0.916	0.504	0.259	0.978

Significant effects (*P* < 0.05) are highlighted in bold.

**Fig 4:**
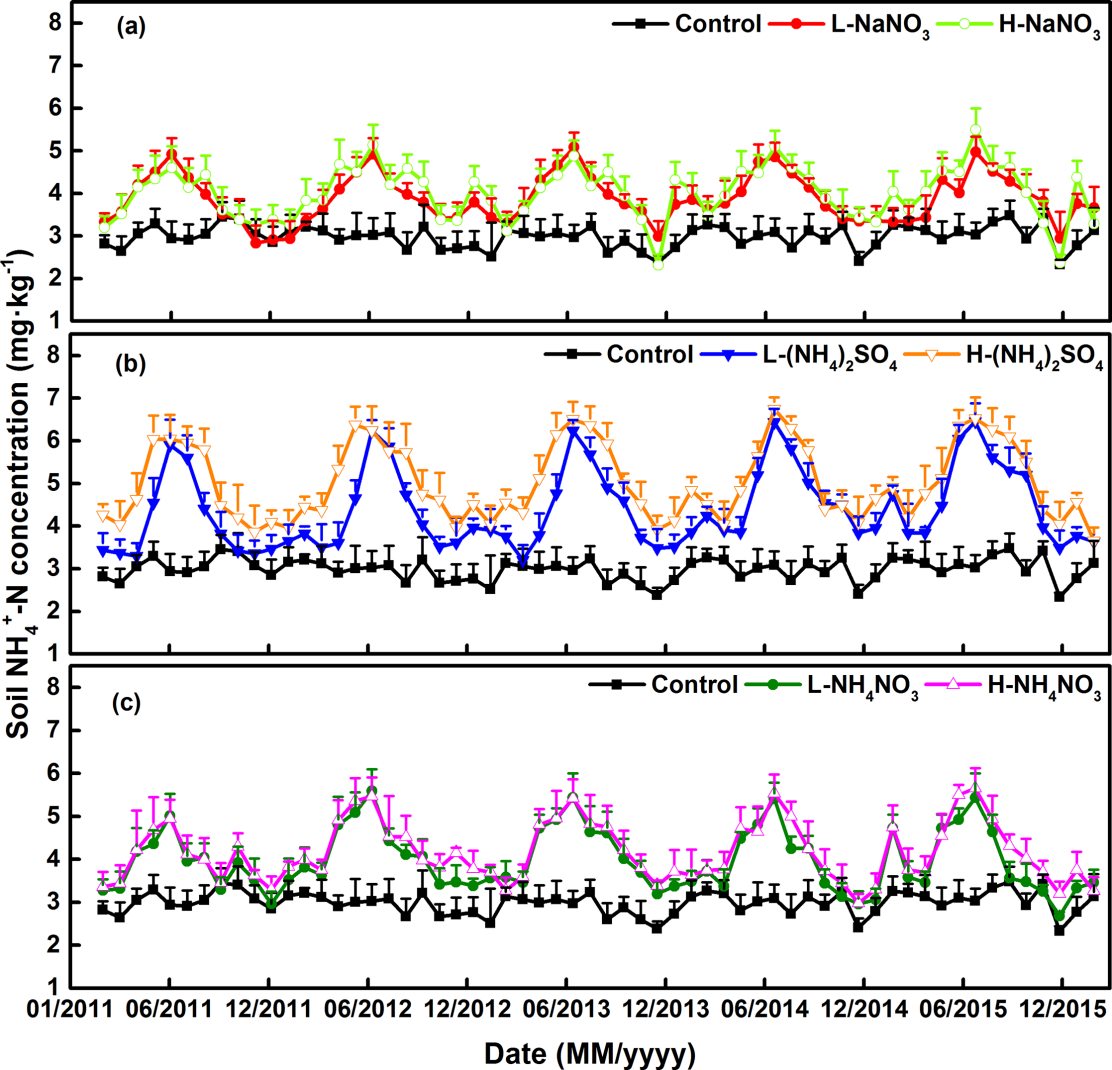
**Variations of soil NH_4_^+^–N concentrations applied with different forms and levels of N addition among five–year experimental period (a) NaNO_3_ addition plots; (b) (NH_4_)_2_SO_4_ addition plots; (c) NH_4_NO_3_ addition plots.** L: 50 kg N ha^–1^ yr^–1^; H: 150 kg N ha^–1^ yr^–1^. Error bars indicate the standard error of the mean (n = 9).

**Fig 5:**
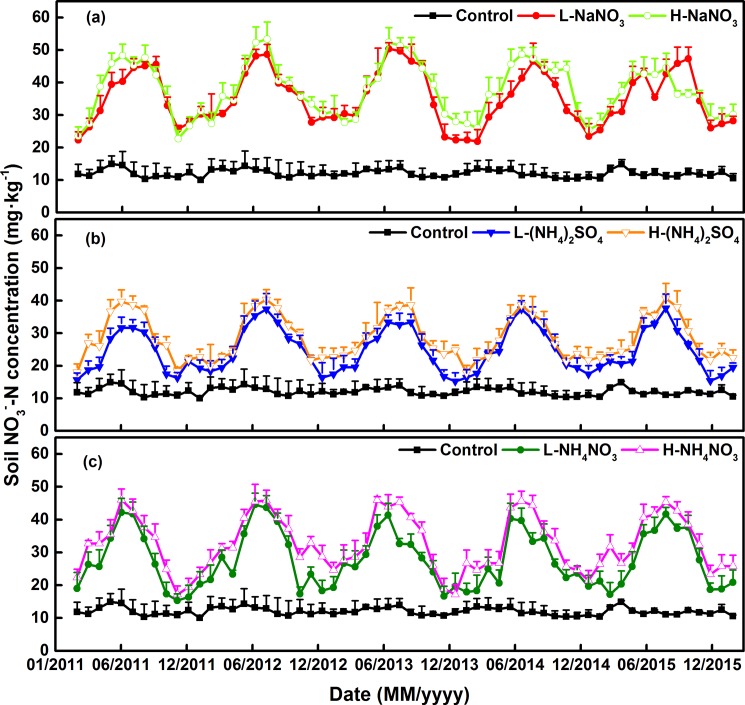
**Variations of soil NO**_**3**_^**–**^**–N concentrations applied with different forms and levels of N addition among five–year experimental period (a) NaNO**_**3**_ **addition plots; (b) (NH**_**4**_**)**_**2**_**SO**_**4**_ **addition plots; (c) NH**_**4**_**NO**_**3**_ **addition plots.** L: 50 kg N ha^–1^ yr^–1^; H: 150 kg N ha^–1^ yr^–1^. Error bars indicate the standard error of the mean (n = 9).

Soil NH_4_^+^–N and NO_3_^–^–N concentrations exhibited significant seasonal variation, with a single peak value in the N addition plots. The maximum value appeared between June and August, while the minimum was observed from November to March (Fig 4, Fig 5).

Soil NO_3_^–^–N accumulated significantly in N addition plots and its concentration ranged from 15.24 to 53.33 mg kg^–1^. N level had a significant promotion effect on soil NO_3_^–^–N concentrations, with those under the and high level N addition had a significantly greater promotion on it compared with that of low level (*P* < 0.05, Table 4). The concentrations from low- and high-level N addition plots were, respectively, 142.14% and 172.90% greater than those from the control (12.03 mg kg^–1^).

Soil NH_4_^+^–N significantly accumulated in the N addition plots and its concentration ranged from 2.32 to 6.74 mg kg^–1^. The accumulation of NH_4_^+^–N caused by N addition was less than that of NO_3_^–^–N in soil. Soil NH_4_^+^–N concentration was significantly influenced by the three N forms, which increased NH_4_^+^–N in the order of (NH_4_)_2_SO_4_ > NH_4_NO_3_ > NaNO_3_ and by 57.40%, 36.27%, and 31.84% when compared with the control (2.98 mg kg^–1^), respectively (Fig 4, Fig 5).

**Fig 6:**
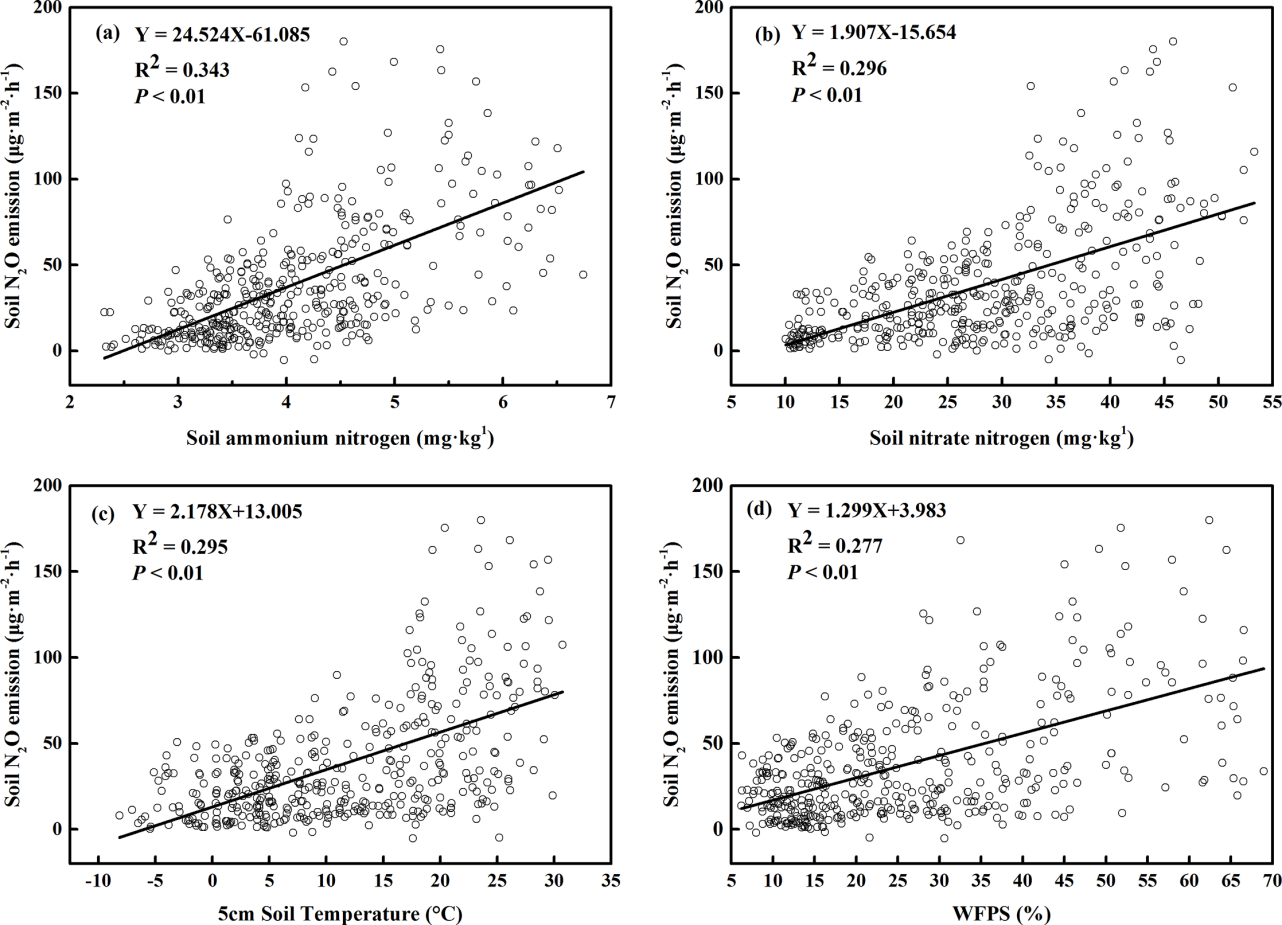
Relationships between soil N_2_O emissions and soil NH_4_^+^–N concentration (a), soil NO_3_^–^–N concentration (b), soil temperature (5 cm depth) (c), WFPS (10 cm depth) (d).

The correlation analysis showed that soil N_2_O emissions were positively correlated with ST at 5 cm depth, WFPS at a 10-cm depth, and soil inorganic nitrogen concentration (Fig 6). In addition, a linear equation showed that soil N_2_O emissions were extremely significantly (*P* < 0.01) correlated with ST, WFPS, and soil NH_4_^+^–N and NO_3_^–^–N (Fig 6).

## Discussion

### Promotion effects of N addition on soil N_2_O emissions

Our results showed that the temperature plantation in northern China was a source of atmospheric N_2_O under natural conditions. The mean N_2_O emissions value in the control was 11.55 μg N_2_O–N m^–2^ h^–1^; this rate is comparable to that reported by Butterbach-Bahl et al. [16] who found that the N_2_O emissions in soils of spruce forests in Germany and Ireland ranged from 3.5 to 16.4 μg N m^–2^ h^–1^. In our study, NaNO_3_, (NH_4_)_2_SO_4_, and NH_4_NO_3_ addition at levels of 50 and 150 kg N ha^–1^ yr^–1^ significantly increased soil N_2_O emissions by an average of 115.26% to 260.15%, 182.92% to 349.77%, 259.89% to 452.02%, respectively. The rate of increase was lower than that for a subtropical forest of the Qianyanzhou Ecological Station, where it was increased by 403% to 762% [5]. Except for L-NaNO_3_ addition, the increase in soil N_2_O emission was higher than the global average (134%) [18]. On one hand, these results indicate that the temperature plantation had high turnover rates of soil N and responded to the increased N deposition. On the other hand, to measure the peak N_2_O emissions in our study, the gas samples were collected in the first, fourth and seventh days after N was added; hence, the cumulative N_2_O emissions might have been overestimated since the N_2_O emissions should have been measured weekly during growing season (to properly reflect an average impact over time). In our previous work, N addition significantly increased the amount of soil microbes and changed the soil microbial community structure in our study area [36]. Soil urease activities were significantly increased by N additions, which promoted soil N_2_O emission [37]. Therefore, the weakened N limitation brought about by a higher litter decomposition rate and greater microbial activity could explain the increased N_2_O emissions we found here [38].

### N_2_O emissions under different N addition forms and levels

Based on our observations over 5 years, the results supported our hypothesis that soil annual cumulative N_2_O emissions increased under elevated N-addition levels. Positive correlations between N-addition levels and soil N_2_O emissions have been found in many previous studies [4, 6, 39–40]. However, at our site, NaNO_3_ addition at a rate of 50 kg N ha^–1^ yr^–1^ did not stimulate a significant increase in the cumulative N_2_O emissions in the first year. Perhaps this is because of a threshold response of soil N_2_O emissions to the N additions [4, 7, 41]. Specifically, such a response is determined by the competition between plants and soil microbes for available N, and thus emissions will not significantly increase until the plant N demands have been satisfied [4, 7, 42].

Considering the addition of different N forms, both NH_4_^+^–N and NO_3_^–^-N significantly promoted soil N_2_O emission and exogenous NH_4_^+^–N and NO_3_^–^-N inputs into our temperate forest had synergic effects on soil N_2_O emission; this result supports our hypothesis and is also consistent with the finding elsewhere that exogenous NH_4_^+^–N and NO_3_^–^–N additions into boreal forest soil can have a synergic effect on its N_2_O emissions [43]. The promotion of NH_4_^+^–N (NH_4_NO_3_ and (NH_4_)_2_SO_4_) additions for N_2_O emission exceeded that provided by the NO_3_^–^–N addition. This result is consistent with other studies finding higher N_2_O emissions from ammonium sources than from nitrate sources [7, 26]. Two potential mechanisms may be responsible for this phenomenon: (1) high immobilization of NO_3_^–^–N and nitrification rates, coupled to a low denitrification potential, led more NO_3_^–^–N to accumulate in soil [44]; (2) poor mobility of NH_4_^+^ created depletion zones around the plant roots, leaving more N input exposed to microorganisms in soils. However, most research to date suggests that denitrification is the main process driving N_2_O production [25, 45]. Yet when WFPS is in the range of 30 to 70%, nitrification can become the main process driving N_2_O production, as denitrification rates increase rapidly when WFPS exceeds 60% [7]. WFPS in our research plots was at a low level for most of the 5-yr monitoring period, only exceeding 60% for a few months, which likely provided less than optimal conditions for the denitrification process [46]. Given this trend in WFPS, we indirectly conclude that NH_4_^+^–N had higher conversion efficiency to N_2_O than NO_3_^–^–N at our forest site.

Although NH_4_^+^–N was always the major N form in local actual N deposition [47], since 1980 its NH_4_^+^–N / NO_3_^–^–N ratio has decreased [48]. Considering the stronger promotion of N_2_O emission by NH_4_^+^, and the decreasing proportion of NH_4_^+^ in N deposition, we expect that the increased soil N_2_O emission stimulated by N deposition at our site will not persist into the future.

### Interannual soil N_2_O emissions under N addition

Considering the time scale, we found a sharp increase in the annual N_2_O emissions in the first three years, but after this point the rate of increase diminished. Soil reaches N saturation when the N input exceeds the N demanded by plants and microorganisms [49]. Early successional forests are always defined as N-limited, because of the limited N availability for vigorous plant growth and the lack of N-fixing plants or bacteria, whereas mature tropical forests and old-growth subtropical forests are typically grouped as being N-saturated [50]. Being N-limited is relative to being N-rich, and this necessarily depends on the soil N availability and the response of vegetation to any N addition [50]. In our study area, N was clearly a limiting factor in the initial years based on amount and stimulating effect of N addition upon tree biomass. Continuing the N addition could shift the soil from being N-limited to N-rich, and then becoming N-saturated, such that soil N_2_O emissions may appear to reach a steady state at high N levels [23]. In addition, Liu and Song [51] found that soil microbial activities may be limited by carbon availability when N is abundant. The suppression of soil N_2_O emissions by long-term N additions was possibly due to a lack of readily available organic carbon [52] and/or adverse effects on mineralization of organic carbon under conditions of high N addition [53]. Therefore, our field experiment highlights the importance carrying out long-term studies to avoid possibly overestimating the N addition effects on N_2_O emissions from short-term observations.

### Relationships between soil N_2_O emissions and soil properties

In our study area, the soil concentration of NO_3_^–^–N was higher than that of NH_4_^+^–N, and the accumulation of NO_3_^–^–N caused by N addition was more than soil NH_4_^+^–N concentration. On the one hand, although the soil NO_3_^–^–N concentration was directly increased by NO_3_^–^–N addition, the NH_4_^+^–N addition could have enhanced the activity of soil nitrifiers and led to the NO_3_^–^–N accumulation in soil we found. This finding and interpretation is consistent with some previous studies carried out in tropical and subtropical forests [54–55]. On the other hand, several studies using the ^15^N tracing method suggest that plants in temperate forest at our site preferred NH_4_^+^–N, which led to more NH_4_^+^–N becoming assimilated, such that the accumulation of NH_4_^+^–N in the soil was relatively little and brief [56].

We found that the soil N_2_O emissions were significantly correlated with concentrations of soil NH_4_^+^–N and NO_3_^–^–N, suggesting soil N_2_O emission was dominated by both nitrification and denitrification processes. Since atmospheric N deposition can significantly promote soil N_2_O emission, and exogenous NH_4_^+^–N and NO_3_^–^-N inputs into temperate forests may have synergic effects on soil N_2_O emission, in the future both of these aspects ought to be distinguished in the dynamics of the N cycle and balance in terrestrial ecosystems by using ^15^N tracer methods. High ST, together with a relatively high WFPS, tend to promote both nitrification and denitrification processes [57] and consequently, high N_2_O emissions, an interpretation that is consistent with many previous findings [58–59]. In particular, high WFPS may promote microbial movement and the expansion of the soil anaerobic microbial community [43]. Warm temperatures benefit soil nitrifying and denitrifying bacteria activities [11], which may explain the seasonal variation in the relatively high N_2_O emissions that occurred from May to September that we observed in this study. Many other complex factors may have a played a role in determining our results, such as soil pH, soil C availability, and the microbial community structure, since they jointly influence the two key processes of nitrification and denitrification that are involved in soil N_2_O production [36, 60].

## Conclusions

This study emphasizes the effects of different N forms and levels on N_2_O emissions from a temperate forest over 5-year experimental period. We found that the accumulation of soil NO_3_^–^-N was significantly higher than that of soil NH_4_^+^–N due to N addition. N addition initially promoted soil N_2_O emission yet this promoting effect, although it existed, weakened in the following years. High level N addition had a stronger promotion effect upon soil N_2_O emission than did the low level N addition. Meanwhile, the combined application of NH_4_^+^–N and NO_3_^–^–N promotes N_2_O emissions more than their single applications, and NH_4_^+^–N addition had a stronger promotion effect for soil N_2_O emission than did the NO_3_^–^–N addition. In addition, WFPS, ST, soil NH_4_^+^–N, and NO_3_^–^–N were all positively related to the N_2_O emissions. In the future, the long-term observation of soil N_2_O emissions, and the measurement of microbial functional groups using ^15^N tracer methods, will be necessary to clarify the mechanisms responsible for the soil N_2_O emissions.

## Supporting information

S1 File Data set underlying the findings. (XLSX)
