# Correction: Five-year study of the effects of simulated nitrogen deposition levels and forms on soil nitrous oxide emissions from a temperate forest in northern China

**DOI:** 10.1371/journal.pone.0196622

**Published:** 2018-04-25

**Authors:** Ke Xu, Chunmei Wang, Xintong Yang

The following information is missing from the Funding section: This study was additionally supported by the National Natural Science Foundation of China (41373069) and Central Universities (2016JX02).
